# Crystal Structure of a Monomeric Thiolase-Like Protein Type 1 (TLP1) from *Mycobacterium smegmatis*

**DOI:** 10.1371/journal.pone.0041894

**Published:** 2012-07-26

**Authors:** Neelanjana Janardan, Rajesh K. Harijan, Rikkert K. Wierenga, Mathur R. N. Murthy

**Affiliations:** 1 Molecular Biophysics Unit, Indian Institute of Science, Bangalore, Karnataka, India; 2 Department of Biochemistry, Biocenter Oulu, University of Oulu, Oulu, Finland; University of Delhi, India

## Abstract

An analysis of the *Mycobacterium smegmatis* genome suggests that it codes for several thiolases and thiolase-like proteins. Thiolases are an important family of enzymes that are involved in fatty acid metabolism. They occur as either dimers or tetramers. Thiolases catalyze the Claisen condensation of two acetyl-Coenzyme A molecules in the synthetic direction and the thiolytic cleavage of 3-ketoacyl-Coenzyme A molecules in the degradative direction. Some of the *M. smegmatis* genes have been annotated as thiolases of the poorly characterized SCP2-thiolase subfamily. The mammalian SCP2-thiolase consists of an N-terminal thiolase domain followed by an additional C-terminal domain called sterol carrier protein-2 or SCP2. The *M. smegmatis* protein selected in the present study, referred to here as the thiolase-like protein type 1 (*Ms*TLP1), has been biochemically and structurally characterized. Unlike classical thiolases, *Ms*TLP1 is a monomer in solution. Its structure has been determined at 2.7 Å resolution by the single wavelength anomalous dispersion method. The structure of the protomer confirms that the N-terminal domain has the thiolase fold. An extra C-terminal domain is indeed observed. Interestingly, it consists of six β-strands forming an anti-parallel β-barrel which is completely different from the expected SCP2-fold. Detailed sequence and structural comparisons with thiolases show that the residues known to be essential for catalysis are not conserved in *Ms*TLP1. Consistent with this observation, activity measurements show that *Ms*TLP1 does not catalyze the thiolase reaction. This is the first structural report of a monomeric thiolase-like protein from any organism. These studies show that *Ms*TLP1 belongs to a new group of thiolase related proteins of unknown function.

## Introduction

The genus Mycobacterium comprises some of the most devastating pathogens that infect both animals and humans. To date, twenty eight genomes of mycobacterial species have been sequenced completely [http://www.ncbi.nlm.nih.gov/genomes/lproks.cgi]. A striking feature in all these genomes is the abundance of genes coding for enzymes involved in fatty acid and lipid metabolism; more than 250 in *Mycobacterium tuberculosis* compared to only 50 in *Escherichia coli*. The mycobacterial genome codes for over a hundred enzymes involved in fatty acid degradation [1]. Apart from providing energy, lipids and fatty acids also form an integral part of the cell wall and cell membrane of Mycobacteria. The abundance and importance of lipid metabolizing enzymes in Mycobacteria make them attractive targets for drug discovery [2], [3]. It is therefore necessary to biochemically and structurally characterize these enzymes.

**Figure 1:**
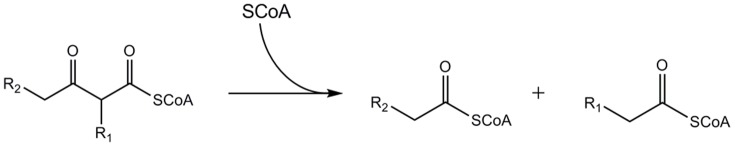
The degradative reaction catalyzed by thiolase. The substrate specificity of different thiolases are distinct: the mammalian SCP2-thiolase accepts as substrate a molecule in which R1 is a 2- methyl group and R2 is a steroid moiety. In all degradative thiolases, the reaction proceeds via a covalent intermediate in which a nucleophilic cysteine is acylated in the first step by the 3-keto substrate, releasing free acetyl CoA (when R1 is H) or propionyl CoA (when R1 is the 2-methyl moiety). The acyl group is subsequently transferred to CoA in the second step of the reaction.

Thiolases are a ubiquitous group of enzymes that are involved in biosynthetic and degradative pathways of lipid metabolism. In the last step of the β-oxidation pathway [4], degradative thiolases catalyze the shortening of the fatty acid chains by degrading 3-keto acyl CoA to form acetyl CoA and a shortened acyl CoA species (Figure 1). Thiolases are a subfamily of the thiolase superfamily. This superfamily also includes the Ketoacyl-(Acyl-carrier-protein) - Synthase (KAS) enzymes, polyketide synthases and chalcone synthases [5], [6], [7]. Most members of this superfamily are dimers and only a few tetramers have been reported. The tetramers are dimers of tight dimers. The best characterized member of the thiolase subfamily is the tetrameric biosynthetic bacterial thiolase from *Zoogloea ramigera* [8], [9]. The key catalytic residue in all characterized members of the thiolase superfamily is a cysteine residue (Cys89 in *Z. ramigera* thiolase). This nucleophilic cysteine participates in the reaction by forming a covalent intermediate with the substrate. There are two other important catalytic residues, either two histidines or an asparagine and a histidine (Asn316 and His348 in *Z. ramigera* thiolase) that are also conserved in the thiolase superfamily [10]. These two residues are important for the formation of an oxyanion hole, which stabilizes the enolate intermediate of the Claisen condensation reaction. The reaction also requires a base to abstract a proton from the substrate. In the biosynthetic thiolase of *Z. ramigera*, a cysteine (Cys378) has been shown to play this role [8]. In thiolases, these catalytic residues are present in four highly conserved loops with characteristic sequence fingerprints [7], [11], [12]. An additional sequence fingerprint near the catalytic site is important for substrate discrimination between unbranched and 2-methyl branched fatty acid tails [7]. Each thiolase subunit is about 400 residues long and has three regions, an N-terminal core region, a loop region (referred to as the thiolase loop domain) and a C-terminal core region [7]. Each of these three regions consists of about 120 residues. The thiolase loop domain shapes the binding pocket of the Coenzyme A (CoA) moiety of the substrate. The N- and C-terminal core regions have similar topologies [12] and appear to be the result of gene duplication. Most of the catalytic residues occur in the C-terminal half but the conserved reactive nucleophile, a cysteine (Cys89 of *Z. ramigera*), is found in the N-terminal half of the protomer. Catalysis in both the synthetic and the degradative directions starts with the acylation of the catalytic, nucleophilic cysteine (Cys89 in *Z. ramigera* thiolase) [8].

In humans, six different thiolases have been identified (CT, T1, T2, TFE, AB and SCP2) [12], [13], [14] with distinct distribution in cellular compartments, quaternary structure, substrate specificity and enzyme kinetics. The sequences of these six thiolases are similar. Of these thiolases, AB [12], T2 [11] and CT [15] have been well characterized and a crystal structure has been determined for each of these enzymes. In contrast, no crystal structures are available for T1, SCP2 and TFE-thiolases. The mammalian SCP2-thiolase has an additional sterol carrier protein C-terminal domain (SCP2) [16]. The structure of SCP2 is known [17], [18].

Examination of the *Mycobacterium smegmatis* genome revealed the presence of several putative thiolase genes [19]. These genes have been annotated as thiolases on the basis of sequence analysis. However, none of them has been biochemically characterized. The sequence identity between some of these proteins and the other well-characterized thiolases is rather low. The protein encoded by one of the *M. smegmatis* thiolase-like genes (the thiolase-like protein type-1, *Ms*TLP1) was over expressed in *E. coli*, purified and crystallized [19]. The crystal structure of this *Ms*TLP1 is described here. Analysis of the structure revealed that the protein has an N-terminal domain with a typical thiolase fold and an additional C-terminal domain. The C-terminal domain is a six stranded anti-parallel β-barrel which is completely different from the structure of SCP2. A detailed analysis of the structure and biochemical properties of this protein revealed that its fold agrees with that of classical thiolase, but the nucleophilic active site cysteine and other characteristic thiolase sequence fingerprints are not conserved. Accordingly, it is found that the protein does not exhibit thiolase activity. Therefore, this protein can be classified as a new subfamily of the thiolase superfamily.

## Results and Discussion

### Bioinformatics analysis

**Figure 2:**
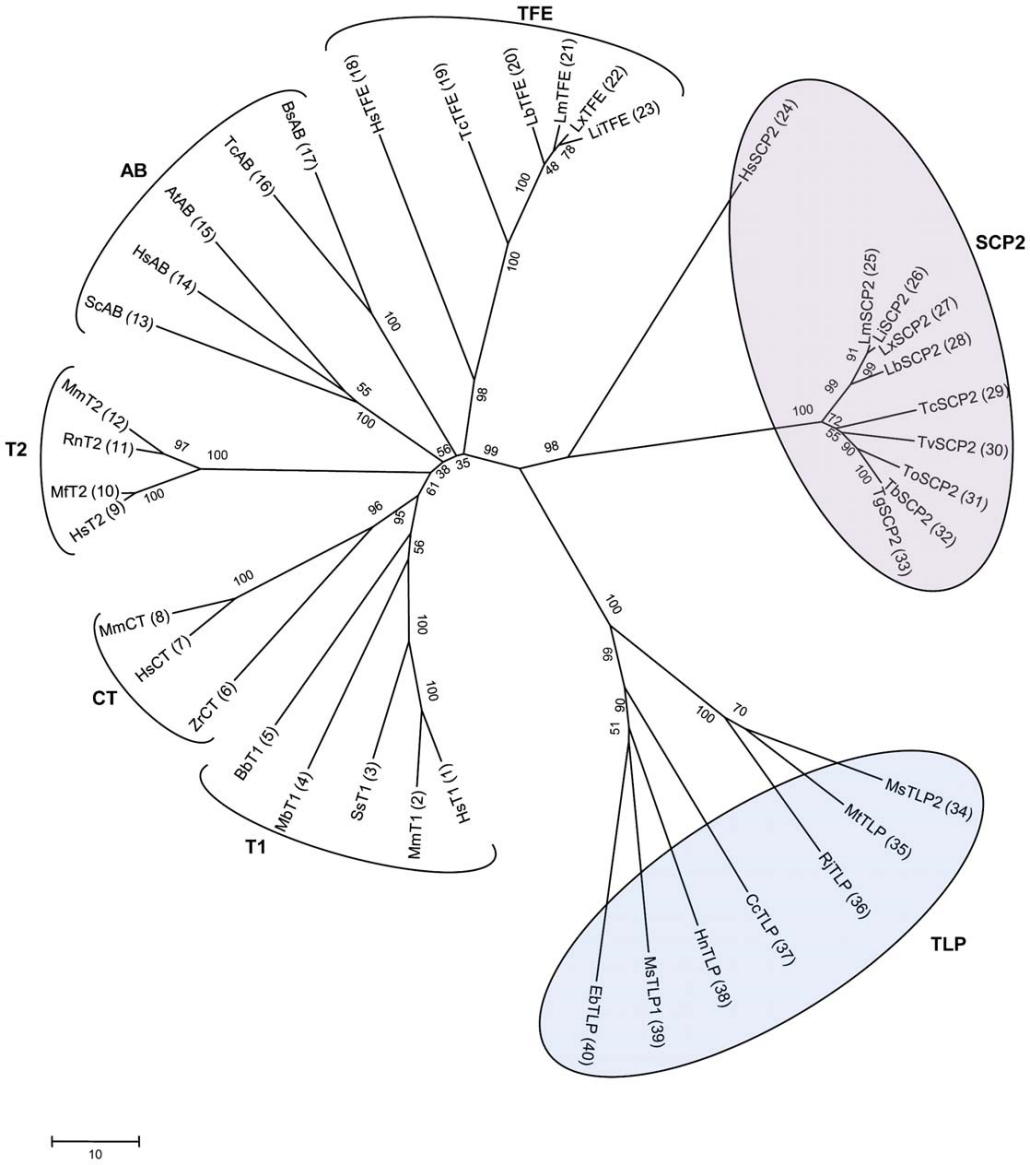
Evolutionary tree analysis of the thiolase sequences. The phylogenetic tree was constructed using the neighbor-joining method, with 10,000 bootstrap replicates in MEGA5 software. Only the region corresponding to the thiolase domain was used for these calculations. The group identifiers correspond to the nomenclature described in the text. The numbers next to each node indicate bootstrap values as percentages. The following sequences (listed with their NCBI accession codes and, for trypanosomatid sequences, their NCBI and GeneDB accession codes) were used for creating the evolutionary tree. **1**, Human: HsT1 (NP_006102.2); **2**, *Mus musculus*: MmT1 (NP_803421.1); **3**, *Salmo salar*: SsT1 (ACI33809.1); **4**, *Monosiga brevicollis*: MbT1 (XP_001748310.1); **5**, *Bdellovibrio bacteriovorus*: BbT1 (NP_967398.1); **6**, *Zoogloea ramigera*: ZrCT (1DM3); **7**, HsCT (NP_005882.2); **8**, MmCT (NP_033364.2); **9**, HsT2 (NP_000010.1); **10**, *Macaca fascicularis*: MfT2 (Q8HXY6.1); **11**, *Rattus norvegicus*: RnT2 (NP_058771.1); **12**, MmT2 (NP_659033.1); **13**, *Saccharomyces cerevisiae*: ScAB (1AFW); **14**, HsAB (NP_001598.1); **15**, *Arabidopsis thaliana*: AtAB (2WU9); **16**, *T. cruzi*: TcAB (Tc00.1047053511003.60, XP_816035.1); **17**, *Bodo saltans*: BsAB (BSA00133, ACI16032.1); **18**, HsTFE (NP_000174.1); **19**, TcTFE (Tc00.1047053511389.150, XP_814301.1); **20**, *L. braziliensis*: LbTFE (LbrM.31.1840, XP_001567180.1); **21**, *L. major*: LmTFE (LmjF.31.1640, XP_001685150.1); **22**, *L. mexicana*: LxTFE (LmxM.30.1640.1, CBZ29221.1); **23**, *L. infantum*: LiTFE (LinJ.31.1660, CAM70518.2); **24**, HsSCP2 (NP_002970.2); **25**, LmSCP2 (LmjF.23.0690, XP_001683404.1); **26**, LiSCP2 (LinJ.23.0860, XP_001465761.1); **27**, LxSCP2 (LmxM.23.0690.1, CBZ27218.1); **28**, LbSCP2 (LbrM.23.0840, XP_001565156.1); **29**, TcSCP2 (Tc00.1047053510507.20, XP_807246.1); **30**, *T. vivax:* TvSCP2 (TvY486_0802010); **31**, *T. congolense*: ToSCP2 (congo1030g10.p1k_8); **32**, *T. brucei*: TbSCP2 (Tb927.8.2540, XP_847087.1); **33**, *T. gambiense*: TgSCP2 (Tbg972.8.2020); **34**, *M. smegmatis*: *Ms*STLP2 (YP_887911.1); **35**, *M. tuberculosis*: MtSTLP (NP_216383.1); **36**, *R. jostii*: RjSTLP (YP_700393.1); **37**, *C. crescentus*: CcSTLP (YP_002517418.1); **38**, *H. neptunium*: HnSTLP (YP_759708.1); **39**, *Ms*TLP1 (YP_889758.1); **40**, *Erythrobacter sp.*: EbSTLP (ZP_01041346.1).

From a genome database search, two TLP coding genes (*M*sTLP1 and *Ms*TLP2) were identified in *M. smegmatis*. The pair wise sequence identity between the two corresponding proteins is 31%. A similar search of the *M. tuberculosis* genome revealed the presence of only one such gene. The pairwise sequence identities of the protein encoded by this gene with *Ms*TLP1 and *Ms*TLP2 are 33% and 69%, respectively. Subsequent searches revealed the presence of TLP homologs in several other bacterial genomes. However, a protein homologous to TLP was not found in the human genome. A multiple sequence alignment was performed using six human thiolases, several other eukaryotic thiolases and seven TLPs from various bacterial sources including *M. smegmatis* and *M. tuberculosis*. The phylogenetic relationships of TLPs with thiolases were analyzed and the results are presented in Figure 2. The TLPs are clustered as a distinct group and the branch point is near the SCP2-thiolase group with high confidence. The mammalian SCP2-thiolase gene codes for an N-terminal thiolase domain and a C-terminal SCP2 domain. Interestingly, the TLP gene also codes for a thiolase like N-terminal domain and an additional C-terminal domain. However, the latter domain is not related to the C-terminal domain of SCP2-thiolase in sequence and its function is yet to be established, as discussed below.

**Figure 3:**
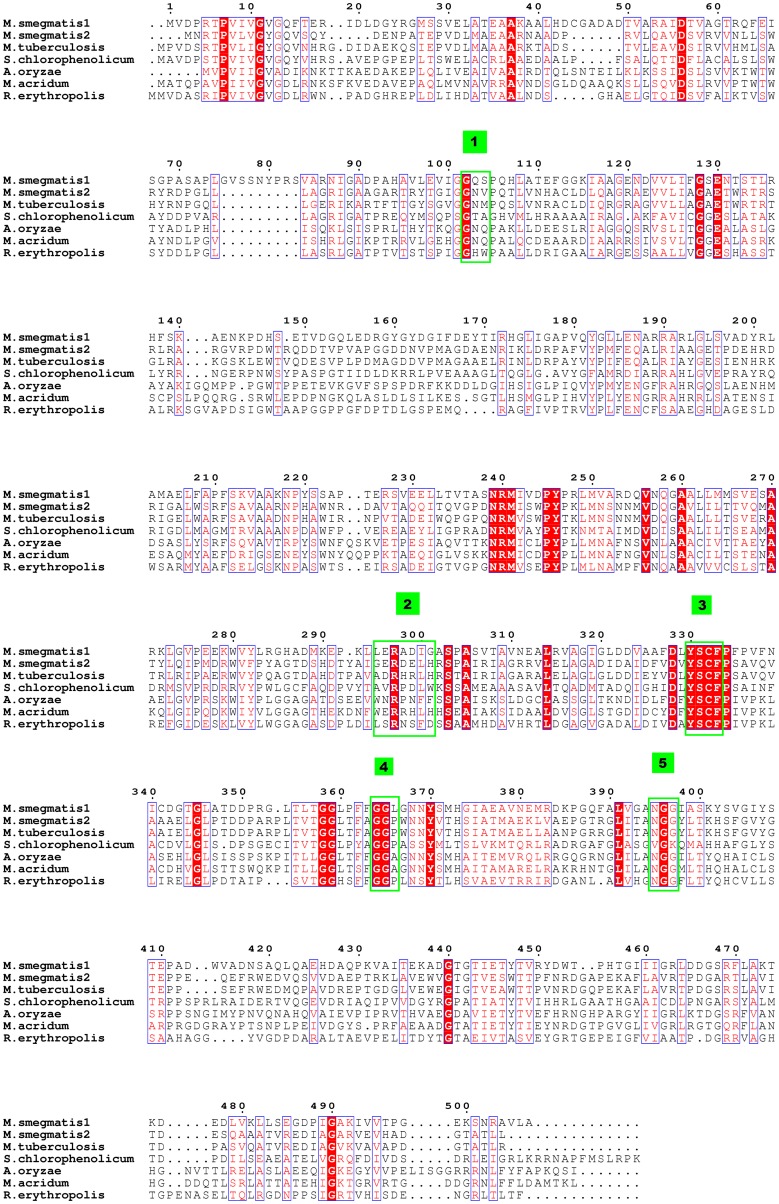
Sequence conservation in TLP proteins. The sequence alignment was achieved using ClustalW program. Seven unique TLP proteins were used for the analysis; TLP1 and TLP2 from *M. smegmatis* (GenBank accession nos. YP_889758.1, YP_887911.1), TLP from *M. tuberculosis* (GenBank accession no. ZP_06454776.1), *S. chlorophenolicum* (GenBank accession no. YP_004555364.1), *A. oryzae*, *M.acridum* (GenBank accession no. XP_001817576.2 ) and *R. erythropolis* (GenBank accession no. ZP_04388669.1). Conserved residues are highlighted in red. Residues structurally equivalent to the five sequence finger prints of classical thiolases are highlighted in numbered green boxes.

**Figure 4:**
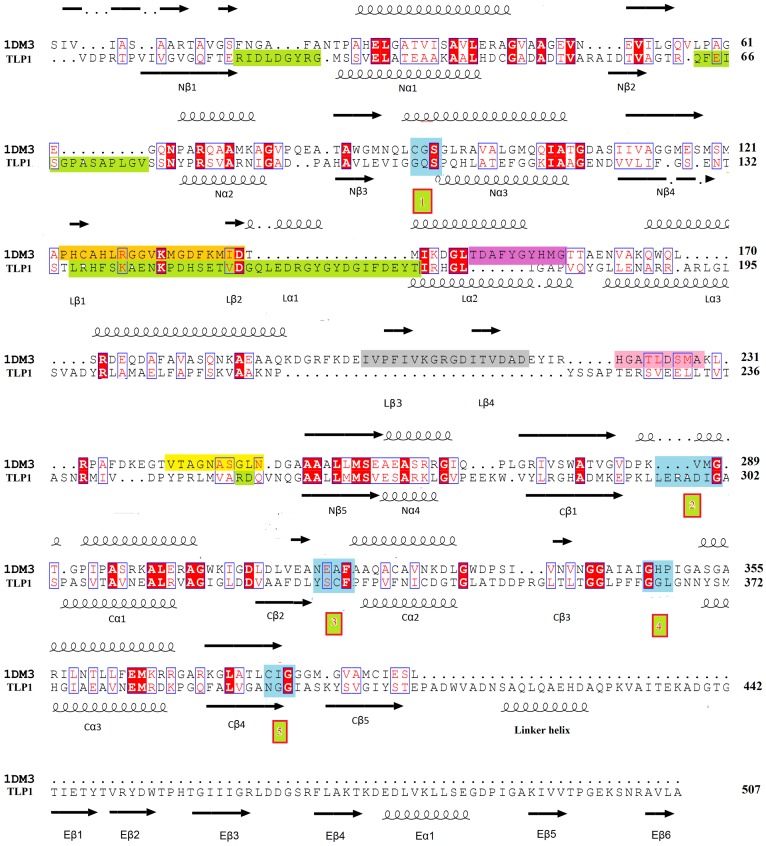
Structure based sequence alignment of *Z. ramigera* thiolase (PDB ID: 1DM3) and *Ms*TLP1 (TLP1, GenBank accession no. YP_889758.1). The secondary structure assignment above and below the sequences refer to *Z. ramigera* thiolase and *Ms*STPL1, respectively. Black coils represent α-helices and black arrows represent β-strands. Residues conserved in both proteins are highlighted in red. The sequence identity between the thiolase domains of the *Z. ramigera* enzyme and *Ms*TLP1 is 15%. The five sequence fingerprints of *Z. ramigera* thiolase and their corresponding residues in *Ms*TLP1 are colored blue, and labeled in green boxes as in Figure 3,. The five segments of the thiolase loop domain with specific functions are highlighted as follows: tetramerization loop (orange), covering loop (purple), cationic loop (grey), adenine loop (light pink), pantetheine loop (yellow). Disordered regions of the *Ms*TLP1 thiolase domain are highlighted in light green.

A multiple sequence alignment reveals the presence of a unique YSCF sequence (residues 330–333 of TLP1) finger print in TLPs (Figure 3). The pair wise, structure based sequence alignment between the *Ms*TLP1 and the bacterial *Z. ramigera* thiolase (PDB code: 1DM3) (Figure 4) shows that this sequence fingerprint corresponds to the NEAF sequence (316–319 of *Z. ramigera* thiolase) motif of thiolases. This motif is known to be important for the geometry of the oxyanion hole [9]. Similarly, four other well characterized, functionally important sequence fingerprints of thiolases (CXS, 89–91; VMG, 287–289; GHP, 347–349 and CXG, 378–380 of *Z. ramigera* enzyme) were also not conserved in *Ms*TLP1 (Figure 4). The sequence identity between MsTLP1 and *Z. ramigera* thiolase is 15% (Figure 4).

### Enzymological characterization

The molecular mass of an *Ms*TLP1 protomer as calculated from the sequence is 56 kDa. Static Light Scattering (SLS) and size exclusion chromatography experiments show that, unlike other thiolases, *Ms*TLP1 is a monomer of 56 kDa in solution (Figure S1). Similarities between the structures of *Ms*TLP1 and thiolases suggest that *Ms*TLP1 might be able to bind CoA and its derivatives. However, thiolase activity could not be detected in either the forward or the reverse direction. The assays were carried out with two different batches of freshly purified enzyme.

Surface Plasmon Resonance (SPR) binding assays showed that TLP1 binds CoA (Figure S2). The binding curves were fitted to the Langmuir binding equation and the affinity of *Ms*TLP1 for CoA was found to be in the millimolar range (K_d_ = 0.6 mM ±0.3) (n = 3). The assays were carried out using two different batches of freshly purified enzyme.

### Structure determination and model quality

The crystal structure of *Ms*TLP1 was determined using the single wavelength anomalous dispersion (SAD) method. As the triclinic P1 cell could accommodate six protomers of the polypeptide, rotation functions were computed using reflections in the 10 Å - 4 Å resolution shell and a radius of integration of 30 Å. Rotation functions corresponding to κ = 180° and κ = 120° hemispheres had significant peaks consistent with non-crystallographic 32 symmetry [19].

**Figure 5:**
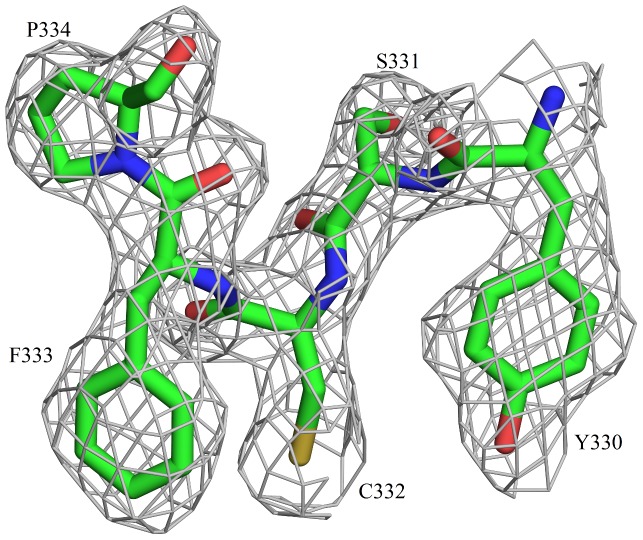
Quality of the final electron density map. Residues 330–334 corresponding to a loop in the N-terminal thiolase domain are shown with the corresponding electron density of the (2F_o_−F_c_), α_c_-map, contoured at 1 σ.

The structure determination was not straightforward and various strategies as described in the methods section had to be used for obtaining a nearly complete structure. About 63 residues of the thiolase domain distributed over four loops could not be built, indicating that these residues belong to truly disordered segments. Electron density is missing for four segments consisting of residues 17–25, 64–77, 135–170 and 253–254 in the thiolase domain (see also Figure 4) as well as for the N-terminal hexa histidine tag. In the rest of the polypeptide, adequate density is not observed for the side chains of only a few surface residues. The data processing and structure refinement statistics are shown in Tables 1 and 2 respectively. The quality of the final electron density map is illustrated in Figure 5. The electron density difference map has an elongated unmodeled feature at an inter-hexamer crystal contact region close the disordered loop 63–76. The uninterpreted density is not connected to the residues at the beginning or end of the disordered loop. The final R-factor and R-free of the refinement at 2.7 Å resolution are 23.3% and 26.1%, respectively (Table 2).

**Table 1:** Data collection statistics for *Mycobacterium smegmatis* TLP1.

	Se-Peak-1	Se-Peak-2	Se-Peak-3
Space group	P1	P1	P1
Wavelength(Å)	0.9789	0.9789	0.9789
Temperature (K)	100	100	100
Resolution range (Å)	50.00-2.60(2.64-2.60)	50.00-2.60(2.64-2.60)	50.0-2.70(2.75-2.70)
Unit cell parameters (Å, °)	a = 96.30, b = 101.95, c = 102.40, α = 116.32, β = 101.20, γ = 97.71	a = 96.30, b = 101.95, c = 102.40, α = 116.32, β = 101.20, γ = 97.71	a = 96.30, b = 101.95, c = 102.40, α = 116.32, β = 101.20, γ = 97.71
Observed reflections	197,597	196,361	172,105
Unique reflections	53,404	38,502	41,976
Redundancy	3.7(2.8)	5.1(3.7)	4.1(2.8)
Mosaicity (deg)	0.7	0.7	0.7
Data completeness (%)	97.1(84.9)	97.3(86.5)	95.3(77.2)
I/σ(I)	16.6(2.3)	18.8(2.5)	12.8(4.6)
R _merge_ (%)	0.08(0.3)	0.09(0.4)	0.09(0.4)
No. of monomers per asymmetric unit	6	6	6

Values in parenthesis refer to the highest resolution shell.

*I* is the integrated intensity and σ (*I*) is the estimated standard deviation of that intensity.

R_merge_ = (Σ_hkl_Σ_i_(|I_i_(hkl)−<I(hkl)>|)/Σ_hkl_ΣI_i_(hkl), where I_i_(hkl) is the intensity of the i^th^ measurement of reflection hkl and <I(hkl)> is its mean.

**Table 2:** Refinement statistics and model quality.

Resolution (Å)	2.7 (2.77-2.70)
R (%)	23.3 (39.6)
Rfree (%)	26.1 (42.8)
No. of atoms	20423
Protein atoms	20211
Solvent atoms	212
Root mean square deviations from ideal values	
Bond length (Å)	0.01
Bond angle ( degrees )	1.9
Average B factors ( Å^2^)	
Main chain atoms	46.3
Side chain atoms and waters	47.1
Residues in Ramachandran plot (%)	
Most favored region	95.6
Allowed region	4.4
Generously allowed region	0.0
Disallowed region	0.0
PDB code entry	4EGV

### The overall fold

**Figure 6:**
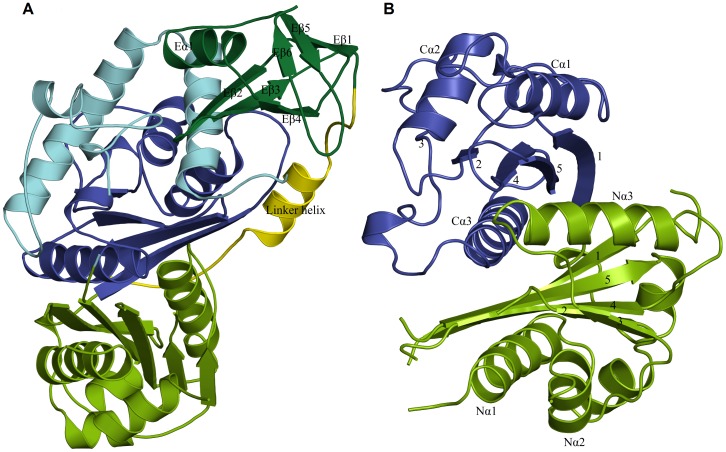
The overall fold of *Ms*TLP1. A) The TLP protomer is divided into two domains. The N-terminal thiolase domain can further be divided into an N-terminal half (green), a thiolase loop region (light blue) and a C-terminal half (dark). The C-terminal extra domain of *Ms*TLP1 is in dark green. The two domains are connected by a linker region (yellow). B) The N-terminal thiolase domain of *Ms*TLP1 has the conserved fold of the thiolase superfamily. The two β sheets are sandwiched between three layers of α helices forming the characteristic α/β/α/β/α layered structure found in classical thiolases. The numbering of the strands and helices conforms to the assignment in the classical thiolases.

The *Ms*TLP1 polypeptide fold is illustrated in Figure 6. The polypeptide folds into two distinct domains: an N-terminal thiolase like domain (residues 1–407) and an additional C-terminal domain (residues 435–507), with a 27 residue long linker (Figure 6A) connecting the two domains. The N-terminal domain of *Ms*TLP1 exhibits the characteristic thiolase fold. DALI search against the PDB using the N-terminal domain as the template revealed several structural homologs. Functionally, all these proteins are involved in fatty acid metabolism. The N-terminal domain can be further divided into two topologically similar N-terminal and C-terminal halves, both of which exhibit the βαβαβαββ topology characteristic of thiolase domains (Figure 6). Between the N- and C-terminal halves is a layer of two α helices contributed by the two halves of the N-terminal domain. The β sheets of the N- and C-terminal halves are also surrounded by a helical layer on the side facing the bulk solvent (Figure 6). Thus, the N-terminal domain is organized into a five layered α/β/α/β/α structure characteristic of the thiolase superfamily. In thiolases, the topologically similar N- and C-terminal halves are connected by the long thiolase loop domain consisting of about 120 residues. In *Ms*TLP1, this region corresponds to residues 133 to 253. The lengths of the loop domains in thiolases and *Ms*TLP1 are comparable. However, there is no electron density for a large segment at the N-terminal region of this loop in *Ms*TLP1 (135–170), indicating that this region of the loop domain is highly flexible. The end of the thiolase loop domain is also disordered in *Ms*TLP1 (Figure 4). Two other disordered loops of the *Ms*TLP1 thiolase domain consisting of 9 and 14 residues, respectively, occur after Nβ1 and Nβ2.

The C-terminal extra domain (residues 435–507) is made up of 6 β strands and resembles a barrel-like structure with three anti-parallel β-strands on either side of the barrel (Figure 6A). There is a short helix located at the top of the barrel resembling a lid. The C-terminal extra domain is connected to the N-terminal thiolase domain by a long loop consisting of a short helix (Figure 6A). The topology of the C-terminal domain is reminiscent of single strand nucleic acid binding proteins. DALI search using this domain against the PDB shows structural similarities to a molybdenum binding protein (PDB id: 1H9K) [20] and a single strand DNA binding protein (PDB id: 3PGZ) (Seattle Structural Genomics Center for Infectious Disease; Unpublished). However, no characteristic DNA binding sequence motif could be identified in the domain.

### Organization of protomers in the crystal asymmetric unit

**Figure 7:**
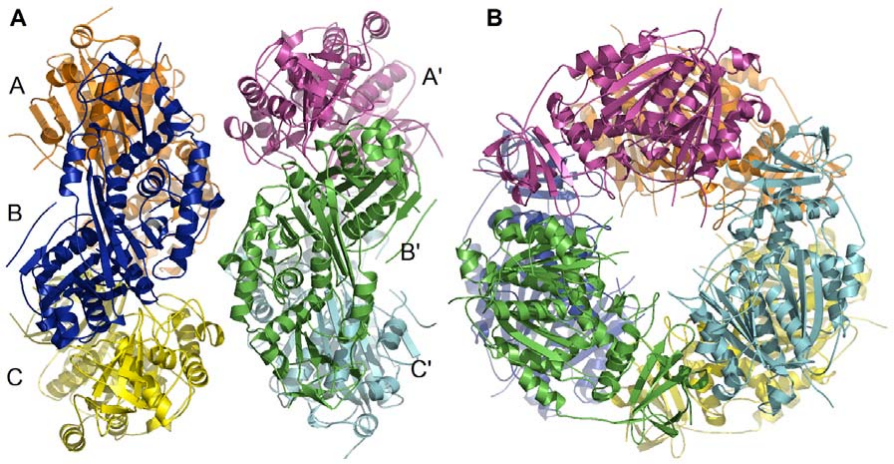
The protomers are assembled with 32 symmetry in the asymmetric unit of *Ms*TLP1. The six protomers are arranged as two discs of three protomers each. A) The two discs are related by a non-crystallographic 2-fold symmetry axis. B) A non-crystallographic 3-fold symmetry axis relates the three protomers of each disk.

The six protomers in the asymmetric unit are related by a nearly perfect non-crystallographic 32 symmetry (Figure 7). The layer of protomers (labeled A, B, C in Figure 7A) related by the non-crystallographic 3-fold symmetry is staggered with respect to the second layer of protomers (labeled A′, B′, C′) related to the first by the non-crystallographic 2-fold symmetry. In the non-crystallographic 3-fold related layer, the inter-protomer contacts are between the thiolase domain of one protomer and the C-terminal domain of the other protomer (Figure 7). The rotational relationship of neighboring protomers related by the non-crystallographic 3-fold axis are within ±0.5° of 120°. Similarly the rotations relating protomers of the two layers are within ±0.5° of 180°.

### Analysis of inter-protomer contacts

The buried surface areas between protomers related by the non-crystallographic 3-fold and 2-fold axes were computed. The solvent accessible surface area of a protomer is 18,145 Å^2^. Due to the 3-fold related interactions, the area that gets buried in the A/B interface is 561 Å^2^ per protomer. This represents only 3.2% of the total area and is small compared to the buried surface area of tightly associated subunits in protein oligomers. This suggests that the interface between 3-fold related subunits is not strong. On the average, about 20 residues from the N-terminal domain of one protomer and 15 residues from the C-terminal domain of the adjacent protomer participate in the interface. The interface is stabilized by 5 hydrogen bonds (Arg 18-Asp 452, Thr 16-Thr 454, Pro 219-Thr 454, Glu 35-Lys 475, Asp 49- Glu 499, Lys 38-Arg 503) and one salt bridge (Glu 35-Lys 473). The contact between protomers related by the non-crystallographic 2-fold is much weaker. No significant hydrogen bonds or salt bridges were detected between the layers. In contrast, an area of 2546 Å^2^ representing 16% of the total surface area gets buried in the dimerization of *Z. ramigera* thiolase. The interface is stabilized by 62 H-bonds and 18 salt bridges and is completely different from the interface between A and A′ subunits of *Ms*TLP1. Therefore, the hexameric state observed in the crystal structure of *Ms*TLP1 is likely to be the result of crystal packing. This observation is consistent with the monomeric state of *Ms*TLP1 in solution suggested by size exclusion chromatography and Static Light Scattering (SLS). Modeling studies suggested that the linker region between the N and C-terminal domains of *Ms*TLP1 (Figure 6A) obstructs the formation of an interface similar to that of *Z. ramigera* thiolase due to steric clashes. Also, the tetramerization loop of *Z. ramigera* thiolase, which occurs at the N-terminal end of the thiolase loop domain (Figure 4), corresponds to a longer, completely disordered region (residues 135–169) in *Ms*TLP1.

### Structural comparison of the thiolase loop domain of *Ms*TLP1 and *Z. ramigera* thiolase

**Figure 8:**
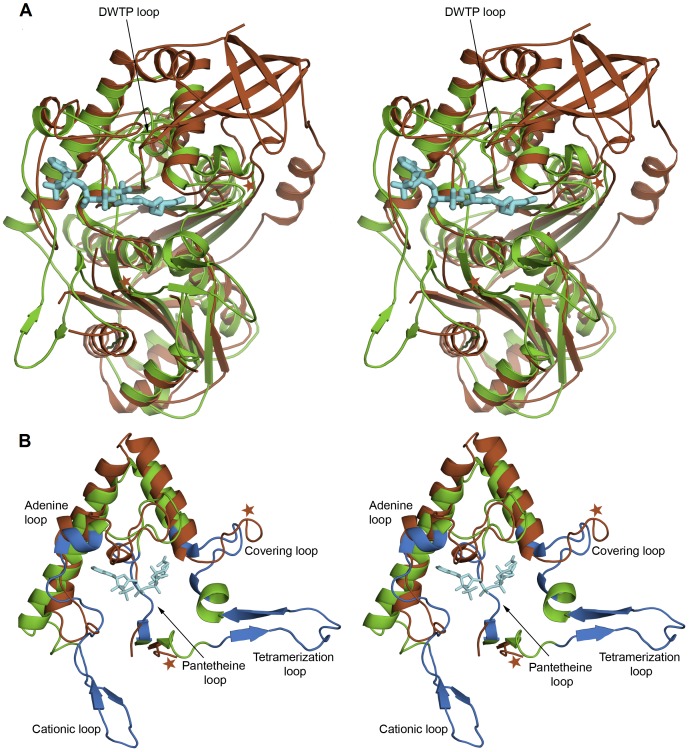
Superposition of *Ms*TLP1 (brown) and *Z. ramigera* thiolase (green). A) Also included is the bound acetyl-CoA (cyan) of the complexed *Z.ramigera* thiolase. It can be noted that the Z. ramigera thiolase CoA-binding part of the thiolase loop domain generates a binding groove in both structures. The DWTP-loop of the C-terminal domain that reaches towards the putative ligand binding site is labeled. The brown stars mark the beginning and the end of the disordered loop (135–170) of MsTLP1 at the beginning of the loop domain. B) Close up of the thiolase loop domains of *Z. ramigera* thiolase (green) and *Ms*TLP1 (brown), highlighting the 5 functional regions (blue) of the *Z. ramigera* thiolase structure.

The most extensively studied thiolase structure is that from *Z. ramigera*. Of the 392 Cα atoms of *Z. ramigera* thiolase, 306 could be superposed on corresponding Cα atoms of the N-terminal domain of *Ms*TLP1 with a root mean square deviation (r.m.s.d.) of 2.4 Å (Figure 8A). It is noteworthy that most of the structural differences observed between *Ms*TLP1 and *Z. ramigera* thiolase are in the thiolase loop domain (119–249 of *Z. ramigera* thiolase). In *Z. ramigera* thiolase, five segments of this domain appear to be important for catalysis and substrate specificity [11], [12]. Figure 8B shows a structural superposition of the thiolase loop domains of *Z. ramigera* thiolase and *Ms*TLP1 highlighting the five functionally important segments. The tetramerization loop occurring at the N-terminus of this domain is essential for the tetrameric organization of *Z. ramigera* thiolase. Residues from this loop also interact with the substrate. The covering loop occurs immediately after the tetramerization loop and covers the active site pocket. The pantetheine loop interacts with the pantetheine part of bound CoA. The covering loop and the pantetheine loop together shape the entrance to the catalytic pocket of Z. *ramigera* thiolase. The cationic loop is solvent-exposed and is thought to capture the negatively charged substrate. The adenine binding loop promotes binding of the adenosine moiety of CoA. The tetramerization loop and the pantetheine loop that occur at the end of the thiolase loop domain are disordered in *Ms*TLP1. From sequence comparison (Figure 4), it is evident that the tetramerization loop is longer in *Ms*TLP1 by about eight residues when compared to the corresponding loop of *Z. ramigera* thiolase. The cationic loop is substantially shortened in *Ms*TLP1 and hence does not resemble the corresponding loop of *Z. ramigera* thiolase. These differences in the conformation of the loops surrounding the active site pocket are likely to be functionally significant. The adenine loop, however, is in a similar position and is of the same length in both the proteins.

### Sequence fingerprints of thiolases

**Figure 9:**
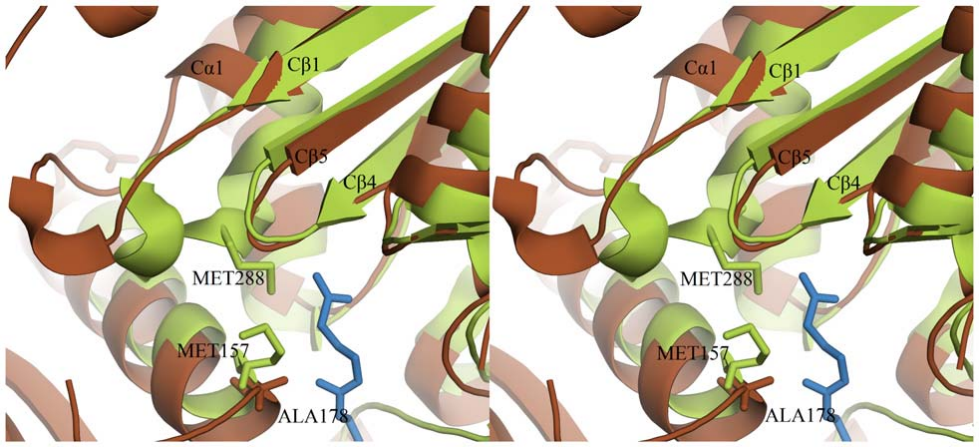
*Ms*TLP1 has a larger binding pocket near its putative catalytic site. In *Z. ramigera* thiolase the size of the pocket is restricted by two methionines (shown in green) that protrude into the binding cavity. In *Ms*TLP1 (brown), one of these residues (Met157 of *Z. ramigera* thiolase) is replaced by Ala178, while the second (Met288 of *Z. ramigera* thiolase) is replaced by a loop containing five residues. However, the loop is displaced away from the cavity.

All enzymes in the thiolase family have five highly conserved sequence fingerprints as shown in Figure 4 [7], [11], [14]. The corresponding residues in *Ms*TLP1 were identified by structure based sequence alignment and careful manual examination (Figure 4). The most significant difference is that the active site nucleophilic cysteine, Cys89 of the CXS motif of *Z. ramigera* thiolase, has been replaced by Gly102 in *Ms*TLP1. This fingerprint is labeled 1 in Figures 3 and 4. The VMG motif (residues 287–289 of thiolase; fingerprint 2) determines the size of the substrate that can be accommodated in the active site cavity. In T2-thiolase [10], the active site cavity can accommodate a branched fatty acid with a 2-methyl group. The inability of *Z. ramigera* thiolase to bind long chain acyl CoA moieties as well as 2-methyl branched fatty acid CoAs has been attributed to the presence of two residues, Met157 and Met288, which protrude into the binding pocket [21], [22]. The *Z. ramigera* thiolase-Met157 has been replaced by a smaller residue Ala178 in *Ms*TLP1. The VMG-motif that includes Met288 is significantly displaced in *Ms*TLP1 such that a much larger volume is available for ligand binding (Figure 9). Therefore, this region of the putative binding pocket of *Ms*TLP1 may bind a more extended ligand. Substantial differences are also observed between *Z. ramigera* thiolase and *Ms*TLP1 in other motifs that define the nature of the active site. Asn316 in the NEAF motif (fingerprint 3; residues 316–319) of *Z. ramigera* thiolase makes a hydrogen bond with the catalytic water [8]. This motif has been replaced by YSCF (residues 330–333) in *Ms*TLP1. The GHP motif (residues 347–349 of *Z. ramigera* thiolase, fingerprint 4), which is involved in the formation of the oxyanion hole, has been replaced by GGL (residues 364–366 of *Ms*TLP1). Finally, Cys379 of the CXG motif (residues 378–380 of *Z. ramigera* thiolase, fingerprint 5), which acts as the base for proton abstraction from the substrate in thiolase, has been replaced by Asn395 of the corresponding NGG motif in *Ms*TLP1 (395–397). The altered motifs YSCF, GGL and NGG are, however, conserved in homologs of *Ms*TLP of other prokaryotes (Figure 3) suggesting that these residues may be important for the function. The conservation of the finger print sequences within TLP homologs and classical thiolases but not between the two families may be a key feature that determines the functional differences between proteins belonging to these families.

### The putative Coenzyme A binding groove of *Ms*TLP1

**Figure 10:**
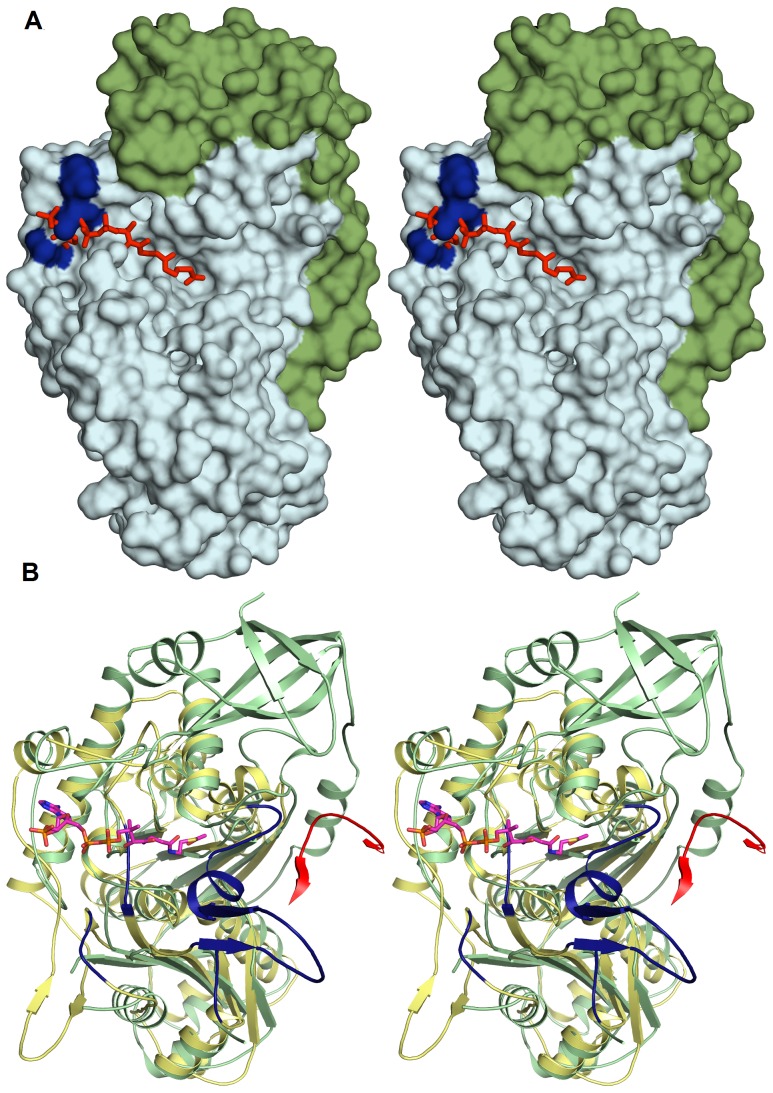
The putative CoA binding groove of *Ms*TLP1 from the comparison with the *Z. ramigera* thiolase. A) The shape of the binding groove. The binding mode of acetyl CoA (red) as obtained by superposition of the complexed *Z. ramigera* thiolase structure. The N-terminal thiolase domain is shown in pale blue and the C-terminal domain is shown in green. Also highlighted in blue are the positively charged residues Arg227, Arg240, and Arg248 which line the putative CoA binding pocket. B). The disordered loops. Loop regions that are disordered in *Ms*TLP1 but ordered in *Z. ramigera* thiolase are highlighted in blue. Also included in the *Z. ramigera* reference structure is the loop just before the dimer interface Nβ3 strand (residues 73–83) of the adjacent subunit (red). This superposition shows that the linker region of *Ms*TLP1 clashes with this loop, thereby preventing the formation of *Z.ramigera* thiolase-like dimers.

The surface characteristics of *Ms*TLP1 are shown in Figure 10A. A large groove is observed extending across the full length of the *Ms*TLP1 molecule. Comparison with the *Z. ramigera* thiolase structure shows that this groove corresponds to the binding site for a CoA molecule or a fatty acyl CoA molecule. As in *Z. ramigera* thiolase, several positively charged residues, notably Arg227, Arg240, Arg248, line this putative CoA-binding pocket, presumably to stabilize binding of the negatively charged phosphate groups of the ligand (Figure 10A). An analysis of the loops of TLP1 (Figure 4) shows that the shape and binding properties of the groove extending beyond the CoA molecule will be affected by the disordered loops occurring at the beginning (135–169) and end (residues 153, 154) of the loop domain, as visualized in Figure 10B. In particular, the disordered loop at the beginning of the loop domain (135–169) is rather long and could cover part of the binding pocket for CoA or a fatty acyl CoA molecule after complex formation. Both loops may become ordered on binding the physiological ligand, thereby shielding the bound ligand from bulk solvent.

### The C-terminal domain

The C-terminal domain of *Ms*TLP1 does not have the same fold as the SCP2 domain of human SCP2-thiolase. The residues of the linker region between the thiolase domain and the C-terminal domain prevent formation of dimers resembling those of classical thiolases. This concerns for example, clashes between the *Ms*TLP1 linker helix (residues 419–427) and the loop region just before the Nβ3 dimer interface β-strand of the other subunit of the hypothetical dimer (residues 73–83), as shown in Figure 10B.

The interface area between the N-terminal thiolase domain and the C-terminal domain is 1035 Å^2^. This constitutes 22% of the C-terminal domain and 4% of the N-terminal domain surface areas, respectively. There are 19 hydrogen bonds and one salt bridge (between Asp487 of the C-terminal domain and Arg190 of the thiolase domain) between the two domains. Interestingly, Arg190 is largely conserved in the homologs of TLP. Asp487 is also conserved in the homologs of TLP. In three of the seven homologs shown in Figure 3, it has been replaced by glutamate and hence the charge is conserved. Thus it appears that most of the TLP homologs have a C-terminal domain anchored to the thiolase domain by similar interactions. This domain is not close to the putative active site, except for residues DWTP (452–455) of the long loop occurring after Eβ1 (Figure 4, Figure 8A). This loop is hydrogen bonded to the thiolase domain via an anti-parallel β-sheet interaction between residues TVR (448–450) and IVD (242–244) of the region just before Nβ5 of the thiolase domain. It is interesting to note that residues of this domain have a slightly higher B-factor (54.3 Å^2^) as compared to the N-terminal thiolase domain (41.6 Å^2^). The role of the C-terminal domain is yet to be elucidated. Deletion studies are underway to shed more light on the functional importance of this domain.

### Concluding remarks

All enzymes of the thiolase superfamily that have been structurally characterized so far share four features: 1) conservation of the core α/β/α/β/α-layered structure of the thiolase domain, 2) conservation of the extensive dimerization interface, 3) the location of the active site pocket and conservation of key active site residues and 4) the use of a nucleophilic cysteine residue in catalysis. *Ms*TLP1 has the conserved core α/β/α/β/α structure and conforms to the thiolase fold strictly. Although the dimerization is not a conserved feature in *Ms*TLP1, the location of the putative active site is similar to those in other thiolases. The ligand binding groove of *Ms*TLP1, identified by structural superposition with *Z. ramigera* thiolase, is larger than that of *Z. ramigera*. The absence of the catalytic cysteine and the consequent lack of thiolase activity suggest that though the protein has the strictly conserved thiolase fold, it might perform an entirely different function. Therefore, the protein appears to belong to a new subfamily in the thiolase superfamily. *Ms*TLP1 is the first monomeric member of this superfamily. The initial sequence annotation suggested correctly that *Ms*TLP1 is a thiolase-like protein, although the sequence identities are not very significant. Interestingly, none of the catalytic residues of the thiolase subfamily is conserved in *Ms*TLP1. Nevertheless, the fold of the thiolase part of *Ms*TLP1 closely resembles the *Z. ramigera* thiolase structure. The location of the ligand binding pocket for CoA in the two proteins also appears to be conserved. *Ms*TLP1 has a unique extra C-terminal domain of unknown function. The structure of this domain is completely different from that of the SCP2 domain of SCP2-thiolase. Examination of TLP1 homologs suggests that the interactions between the thiolase-like domain and the C-terminal additional domains are largely conserved. The physiological function of the full length *Ms*TLP1 protein has not yet been established. Further studies have been initiated to establish the intriguing role of *Ms*TLP1 in the lipid metabolism of mycobacterium and other microorganisms.

## Materials and Methods

### Cloning, expression and purification

The gene coding for *M. smegmatis* TLP1 (YP_889758) was cloned, over-expressed in *E. coil* and purified as described earlier (19).

### Selenomethionine incorporation

The *Ms*TLP1 clone was transformed into BL21 (DE3) Rosetta strain of *E. coli*. A single colony was picked and grown in 1 ml LB-ampicillin overnight at 37°C. This was then inoculated into 500 ml minimal media with 100 µg/ml ampicillin and grown for 12 hrs at 37°C. Methionine synthesis was inhibited by the addition of an amino acid mixture containing 50 mg/l of leucine, isoleucine, valine, lysine, threonine and phenylalanine. After incubation for half an hour, protein expression was induced with 1.0 mM isopropyl-beta-thio galactopyranoside (IPTG). Selenomethionine was added at the time of induction. Purification of the protein was carried out as described for the native enzyme. Selenomethionine incorporation was confirmed by accurate mass determination using Electrospray ionisation mass spectrometry (ESI-MS).

### Crystallization, data collection and data processing

The crystals were obtained as described previously [19]. Briefly, the crystals were grown at room temperature using the microbatch method. A crystallization droplet consisted of 2 µl of protein solution (5 mg/ml in 50 mM Tris-HCl pH 8.0 containing 100 mM NaCl, 10% glycerol, 5 mM 2-mercaptoethanol) and 2 µl of crystallization solution (100 mM 4-(2-hydroxyethyl)-1-piperazineethanesulfonic acid (HEPES) pH 7.5, 20% Polyethylene glycol (PEG) 4000, 10% isopropanol). Selenium incorporated *Ms*TLP1 crystals were obtained under the same conditions. Three data sets of selenomethionine labeled crystals (Se-*Ms*TLP1) were collected to 2.7 Å resolution at a wavelength of 0.9789 Å (peak) at beam line BM14 of the European Synchrotron Radiation Facility (ESRF), Grenoble, France, using three different crystals. The data were processed using DENZO and SCALEPACK from the HKL-2000 suite [23]. Data processing revealed that the crystal belonged to the triclinic space group P1 and the size of the unit cell is compatible with 4–8 protomers of MW 56 kDa corresponding to Matthews coefficients of 4.0 Å^3^Da^−1^−2.0 Å^3^Da^−1^, respectively. The final statistics for data collection and processing are summarized in Table 1.

### Structure solution and refinement

The structure of *Ms*TLP1 was determined as follows. Using anomalous differences, 63 selenium positions were identified and refined using the program SHELXD [24]. Initial selenium position based phases obtained by the PHENIX program were improved by solvent flattening using the program DM of CCP4 [25] suite. The resultant map was used for automated model building with the program Autobuild of the Phenix suite [26]. The first round of automated model building could generate only 40% of the structure. The model obtained was manually adjusted using the interactive graphics program COOT [27] and refined using REFMAC5 [28] of the CCP4 suite. Subsequent rounds of manual model building and refinement extended the structure by an additional 20%. At this stage, a phase combination of the initial phases obtained by anomalous data and the model based phases was carried out using the program Phase-Combine of CCP4 suite [25]. The electron density map obtained using the combined phases was used for a new round of automated model building. This extended the structure by an additional 5%. At this stage, an anomalous difference Fourier map was calculated and selenium positions were re-determined. The 62 highest peaks in the difference Fourier map were accepted as representing selenium atoms. Between this set and the earlier set of selenium positions, 50 were common. The new selenium positions were subjected to extensive refinement using PHASER [29]. Phases obtained using these positions were used for map calculation and automated model building. At this stage, 80% of the structure could be built.

These calculations were based on only one of the three data sets collected at the peak wavelength. At this stage, all the three peak data sets collected at ESRF were merged and scaled and the resulting data were used to perform Molecular Replacement- Single wavelength Anomalous Dispersion (MR-SAD) using PHASER [29] along with the 80% model already built as the partial structure for initial phase calculation. This extended the model by an additional 10% and brought the structure to its current state of completion, i.e. 90%.

### Structure analysis

The geometry of the final model was examined using PROCHECK [30]. All structural superpositions were achieved using the SSM superpose feature of COOT [27]. Average B-factors for protein atoms, water molecules and ligands were calculated using BAVERAGE of the CCP4 suite. The PISA [31] server was used for interface area calculations. Surface area calculations and analysis of contacts were performed using programs available in the CCP4 suite [25]. All figures were prepared using PYMOL (The PyMOL Molecular Graphics System, Version 1.2r3pre, Schrödinger, LLC). Bacterial *Z. ramigera* thiolase (PDB ID: 1DM3) has been used for all sequence and structural alignments. 1DM3 represents the acetylated *Z. ramigera* thiolase structure complexed with acetyl CoA.

### Bioinformatics analysis

Only the thiolase domain of *Ms*TLP1 was used in all sequence analyses. The amino acid sequence of *Ms*TLP1 was used as a starting point in an extensive bioinformatics analysis of the *M. smegmatis* genome: (i) Proteins homologous to *Ms*TLP1 were identified in the non-redundant protein sequence database of SWISSPROT [32] using BLAST [33]. (ii) Additional sequences that might be related to *Ms*TLP were identified using the six characterized human thiolases as query sequences in the BLAST program. Two TLP homologs, TLP1 and TLP2 (YP_887911.1 and YP_889758.1), were identified in the *M. smegmatis* genome. The catalytic cysteine is not conserved in either of these proteins although they have been annotated as thiolases. These proteins appear to have an N-terminal thiolase-like domain and an extra domain at the C-terminus. (iii) A BLAST search against the human genome using the *Ms*TLP1 amino acid sequence did not reveal any hits. However, homologs of *Ms*TLP1 could be identified in many prokaryotic genomes. TLP homologs were also observed to be present in all other *Mycobacterium* spp. Interestingly, only one TLP sequence was found in the *M. tuberculosis* genome. (iv) A multiple sequence alignment was performed by ClustalW [34] using amino-acid sequences of the six human thiolases, several other eukaryotic thiolases and seven TLPs from various sources including two *mycobacterial spp*. This alignment was used to generate a phylogenetic tree using the neighbor-joining method with 10,000 bootstrap replicates in MEGA5 [35].

### Oligomeric state of *Ms*TLP1

The oligomeric state of *Ms*TLP1 in solution was determined using a static light scattering (SLS) system (Wyatt Minidawn) with its flow cell connected to an ÄKTA purifier operated at 17°C. *Ms*TLP1 (0.5 ml of 4.0 mg/ml) protein solution in 50 mM Tris-HCl pH 8.0, 100 mM NaCl, 10% glycerol, 5 mM 2-mercaptoethanol was loaded on a Superdex 200 (10/300) GL column (GE Healthcare) attached to the ÄKTA purifier. The elution profiles (Supplementary Figure S1) were also monitored using RI and UV detectors and analysed using the ASTRA program. The molecular mass expected from the sequence was calculated using the online tool PROTPARAM [36].

### Thiolase activity assays

The thiolase activity in the degradative (Figure 1) and synthetic directions were estimated as described previously [8] at 30°C using a Shimadzu UV-1800 299 spectrophotometer. The concentrations of the substrates CoA, acetyl CoA and acetoacetyl CoA, in the respective stock solutions, were verified using the Ellman's test [37]. In all the assays, a cocktail without *Ms*TLP1 served as the negative control and measurements with active trypanosomal thiolase were used as the positive control.

In the thiolytic direction, the 500 µl reaction cocktail contained 50 mM Tris-HCl pH 7.8, 25 mM MgCl_2_, 60 µM CoA and 50 µM acetoacetyl CoA. The disappearance of the Mg^2+^/acetoacetyl CoA complex was measured at 303 nm for 3 min after the addition of 10 µg of *Ms*TLP1. In the synthetic direction, the activity assay was carried out using a short chain 3-hydroxyacyl CoA dehydrogenase (SC-HAD) as the linker enzyme [9]. In this assay, the formation of acetoacetyl CoA is measured by incubating the enzyme with acetyl CoA and measuring the product formation by reducing it with nicotinamide adenine dinucleotide (NADH), as catalyzed by the linker enzyme (as described previously [11]). The reaction was initiated by the addition of 10 µg of *Ms*TLP1 to a mixture containing 50 mM Tris-HCl, pH 7.8, 40 mM KCl, 0.2 mM NADH, 1 U of SCHAD (1 U is 1 µmol/(min. mg) of protein), 0.5 mM dithiothreitol (DTT) and 2 mM acetyl CoA. The total reaction volume was 500 µl and the rate of NADH oxidation was monitored at 340 nm for 3 minutes.

### CoA affinity studies

The binding studies between *Ms*TLP1 with CoA were performed with a Biacore 3000 (Biacore, Uppsala, Sweden) optical biosensor at 25°C. 2 µM of *Ms*TLP1 protein was immobilized by amine coupling on the surface of a CM5 sensor chip (GE Healthcare) to a level of ∼3,000 response units using the GE Healthcare standard immobilization protocol. Binding and dissociation were measured at a flow rate of 20 µl per minute. The ligand solution was run over the sensor surface at five different ligand concentrations (0.3, 0.6, 1.25, 2.50 and 5 mM) in a running buffer of 10 mM HEPES pH 7.4, 150 mM NaCl, 3 mM ethylenediaminetetraacetic acid (EDTA) and 0.005% P-20 The sensor surface was regenerated with borate buffer pH 8.5. The binding curves were corrected for non-specific binding by subtracting the signal obtained for the negative control flow cell. Kinetic constants for association and dissociation were derived from linear transformations of the binding data. The data were fit to the simple 1∶1 Langmuir interaction model using the BIA EVALUATION software.

## Supporting Information

Figure S1 **The oligomeric state analysis of the recombinantly expressed, purified** ***Ms*** **TLP1 has been determined by using static light scattering (SLS) in combination with size-exclusion chromatography.** A Superdex 200 10/300 GL column (GE Healthcare) was used for the size-exclusion chromatography. The elution profile is provided by the UV signal (thin red line). The SLS signal is combined with the RI-signal for the calculation of the molar mass (thick red line) by the ASTRA program.
(TIFF)

Figure S2 **Kinetics of binding of CoA to** ***Ms*** **TLP1 determined using SPR.** The SPR sensogram was obtained by flowing different CoA solutions (see inset) over the TLP1 immobilized sensor chip. The data show that CoA binds to TLP1 with a K_d_ in the millimolar range.
(TIFF)

## References

[1] Cole ST, Brosch R, Parkhill J, Garnier T, Churcher C, et al (1998) Deciphering the biology of Mycobacterium tuberculosis from the complete genome sequence. Nature 393: 537–544. 9634230 10.1038/31159

[2] Nesbitt NM, Yang X, Fontan P, Kolesnikova I, Smith I, et al (2010) A thiolase of Mycobacterium tuberculosis is required for virulence and production of androstenedione and androstadienedione from cholesterol. Infect Immun 78: 275–282. 19822655 10.1128/IAI.00893-09 PMC2798224

[3] Cole ST, Riccardi G (2011) New tuberculosis drugs on the horizon. Curr Opin Microbiol 14: 570–576. 21821466 10.1016/j.mib.2011.07.022

[4] Hiltunen JK, Qin Y (2000) beta-oxidation - strategies for the metabolism of a wide variety of acyl-CoA esters. Biochim Biophys Acta 1484: 117–128. 10760462 10.1016/s1388-1981(00)00013-5

[5] Heath RJ, Rock CO (2002) The Claisen condensation in biology. Nat Prod Rep 19: 581–596. 12430724 10.1039/b110221b

[6] Austin MB, Noel JP (2003) The chalcone synthase superfamily of type III polyketide synthases. Nat Prod Rep 20: 79–110. 12636085 10.1039/b100917f

[7] Haapalainen AM, Merilainen G, Wierenga RK (2006) The thiolase superfamily: condensing enzymes with diverse reaction specificities. Trends Biochem Sci 31: 64–71. 16356722 10.1016/j.tibs.2005.11.011

[8] Williams SF, Palmer MA, Peoples OP, Walsh CT, Sinskey AJ, et al (1992) Biosynthetic thiolase from *Zoogloea ramigera*. Mutagenesis of the putative active-site base Cys-378 to Ser-378 changes the partitioning of the acetyl S-enzyme intermediate. J Biol Chem 267: 16041–16043. 1353760

[9] Merilainen G, Poikela V, Kursula P, Wierenga RK (2009) The thiolase reaction mechanism: the importance of Asn316 and His348 for stabilizing the enolate intermediate of the Claisen condensation. Biochemistry 48: 11011–11025. 19842716 10.1021/bi901069h

[10] Jiang C, Kim SY, Suh DY (2008) Divergent evolution of the thiolase superfamily and chalcone synthase family. Mol Phylogenet Evol 49: 691–701. 18824113 10.1016/j.ympev.2008.09.002

[11] Haapalainen AM, Merilainen G, Pirila PL, Kondo N, Fukao T, et al (2007) Crystallographic and kinetic studies of human mitochondrial acetoacetyl-CoA thiolase: the importance of potassium and chloride ions for its structure and function. Biochemistry 46: 4305–4321. 17371050 10.1021/bi6026192

[12] Mathieu M, Zeelen JP, Pauptit RA, Erdmann R, Kunau WH, et al (1994) The 2.8 A crystal structure of peroxisomal 3-ketoacyl-CoA thiolase of Saccharomyces cerevisiae: a five-layered alpha beta alpha beta alpha structure constructed from two core domains of identical topology. Structure 2: 797–808. 7812714 10.1016/s0969-2126(94)00081-6

[13] Mazet M, Harijan RK, Kiema TR, Haapalainen AM, Morand P, et al (2011) The characterization and evolutionary relationships of a trypanosomal thiolase. Int J Parasitol 41: 1273–1283. 21907205 10.1016/j.ijpara.2011.07.009

[14] Fukao T, Song XQ, Mitchell GA, Yamaguchi S, Sukegawa K, et al (1997) Enzymes of ketone body utilization in human tissues: protein and messenger RNA levels of succinyl-coenzyme A (CoA):3-ketoacid CoA transferase and mitochondrial and cytosolic acetoacetyl-CoA thiolases. Pediatr Res 42: 498–502. 9380443 10.1203/00006450-199710000-00013

[15] Kursula P, Sikkila H, Fukao T, Kondo N, Wierenga RK (2005) High resolution crystal structures of human cytosolic thiolase (CT): a comparison of the active sites of human CT, bacterial thiolase, and bacterial KAS I. J Mol Biol 347: 189–201. 15733928 10.1016/j.jmb.2005.01.018

[16] Seedorf U, Brysch P, Engel T, Schrage K, Assmann G (1994) Sterol carrier protein X is peroxisomal 3-oxoacyl coenzyme A thiolase with intrinsic sterol carrier and lipid transfer activity. J Biol Chem 269: 21277–21283. 8063752

[17] Choinowski T, Hauser H, Piontek K (2000) Structure of sterol carrier protein 2 at 1.8 A resolution reveals a hydrophobic tunnel suitable for lipid binding. Biochemistry 39: 1897–1902. 10684638 10.1021/bi992742e

[18] Haapalainen AM, van Aalten DM, Merilainen G, Jalonen JE, Pirila P, et al (2001) Crystal structure of the liganded SCP-2-like domain of human peroxisomal multifunctional enzyme type 2 at 1.75 A resolution. J Mol Biol 313: 1127–113. 11700068 10.1006/jmbi.2001.5084

[19] Janardan N, Paul A, Harijan RK, Wierenga RK, Murthy MR (2011) Cloning, expression, purification and preliminary X-ray diffraction studies of a putative Mycobacterium smegmatis thiolase. Acta Crystallogr Sect F Struct Biol Cryst Commun 67: 817–82. 10.1107/S1744309111019324 PMC3144804 21795802

[20] Delarbre L, Stevenson CEM, White DJ, Mitchenall LA, Pau RN, Lawson DM (2001) Two crystal structures of the cytoplasmic molybdate-binding protein ModG suggest a novel cooperative binding mechanism and provide insights into ligand-binding specificity. J Mol Biol 308: 1063. 11352591 10.1006/jmbi.2001.4636

[21] Modis Y, Wierenga RK (1999) A biosynthetic thiolase in complex with a reaction intermediate: the crystal structure provides new insights into the catalytic mechanism. Structure 7: 1279–1290. 10545327 10.1016/s0969-2126(00)80061-1

[22] Merilainen G, Schmitz W, Wierenga RK, Kursula P (2008) The sulfur atoms of the substrate CoA and the catalytic cysteine are required for a productive mode of substrate binding in bacterial biosynthetic thiolase, a thioester-dependent enzyme. FEBS J 275: 6136–6148. 19016856 10.1111/j.1742-4658.2008.06737.x

[23] Otwinowsky Z, Minor W (1997) Processing of X-ray diffraction data collected in oscillation mode. Methods Enzymol 276: 307–326. 10.1016/S0076-6879(97)76066-X 27754618

[24] Schneider TR, Sheldrick GM (2002) Substructure solution with SHELXD. Acta Crystallogr D Biol Crystallogr 58: 1772–1779. 12351820 10.1107/s0907444902011678

[25] Collaborative Computational Project (1994) The CCP4 suite: programs for protein crystallography. Acta Crystallogr D Biol Crystallogr 50: 760–763. 15299374 10.1107/S0907444994003112

[26] Adams PD, Afonine PV, Bunkoczi G, Chen VB, Davis IW, et al (2010) PHENIX: a comprehensive Python-based system for macromolecular structure solution. Acta Crystallogr D Biol Crystallogr 66: 213–221. 20124702 10.1107/S0907444909052925 PMC2815670

[27] Emsley P, Lohkamp B, Scott WG, Cowtan K (2010) Features and development of Coot. Acta Crystallogr D Biol Crystallogr 66: 486–501. 20383002 10.1107/S0907444910007493 PMC2852313

[28] Murshudov GN, Vagin AA, Dodson EJ (1997) Refinement of macromolecular structures by the maximum-likelihood method. Acta Crystallogr D Biol Crystallogr 53: 240–255. 15299926 10.1107/S0907444996012255

[29] McCoy AJ, Grosse-Kunstleve RW, Adams PD, Winn MD, Storoni LC, et al (2007) Phaser crystallographic software. J Appl Crystallogr 40: 658–674. 19461840 10.1107/S0021889807021206 PMC2483472

[30] Laskowski RA, McArthur MW, Moss DS, Thornton JM (1993) PROCHECK: a program to check the stereo-chemical quality of protein structures. J Appl Crystallog 26: 283–291.

[31] Krissinel E, Henrick K (2007) Inference of macromolecular assemblies from crystalline state. J Mol Biol 372: 774–797. 17681537 10.1016/j.jmb.2007.05.022

[32] The UniProt Consortium (2011) Ongoing and future developments at the Universal Protein Resource. Nucleic Acids Res 39: 214–219. 10.1093/nar/gkq1020 PMC3013648 21051339

[33] Altschul SF, Gish W, Miller W, Myers EW, Lipman DJ (1990) Basic local alignment search tool. J Mol Biol 215: 403–410. 2231712 10.1016/S0022-2836(05)80360-2

[34] Thompson JD, Gibson TJ, Higgins DG (2002) Multiple sequence alignment using ClustalW and ClustalX. Curr Protoc Bioinformatics Chapter 2: Unit 2 3. 10.1002/0471250953.bi0203s00 18792934

[35] Tamura K, Peterson D, Peterson N, Stecher G, Nei M, et al (2011) MEGA5: molecular evolutionary genetics analysis using maximum likelihood, evolutionary distance, and maximum parsimony methods. Mol Biol Evol 28: 2731–2739. 21546353 10.1093/molbev/msr121 PMC3203626

[36] Gasteiger E, Hoogland C, Gattiker A, Duvaud S, Wilkins MR, et al (2005) Protein Identification and Analysis Tools on the ExPASy Server. (In) The Proteomics Protocols Handbook, Humana Press. Walker John M, editor. 571–607.

[37] Riddles PW, Blakeley RL, Zerner B (1983) Reassessment of Ellman's reagent. Methods Enzymol 91: 49–60. 6855597 10.1016/s0076-6879(83)91010-8

