# Supplementary figures and images for: Crystal Structure of a Monomeric Thiolase-Like Protein Type 1 (TLP1) from Mycobacterium smegmatis
Source: PLoS One. 2012 Jul 26;7(7):e41894. doi: 10.1371/journal.pone.0041894 (PMC3406046; doi:10.1371/journal.pone.0041894)

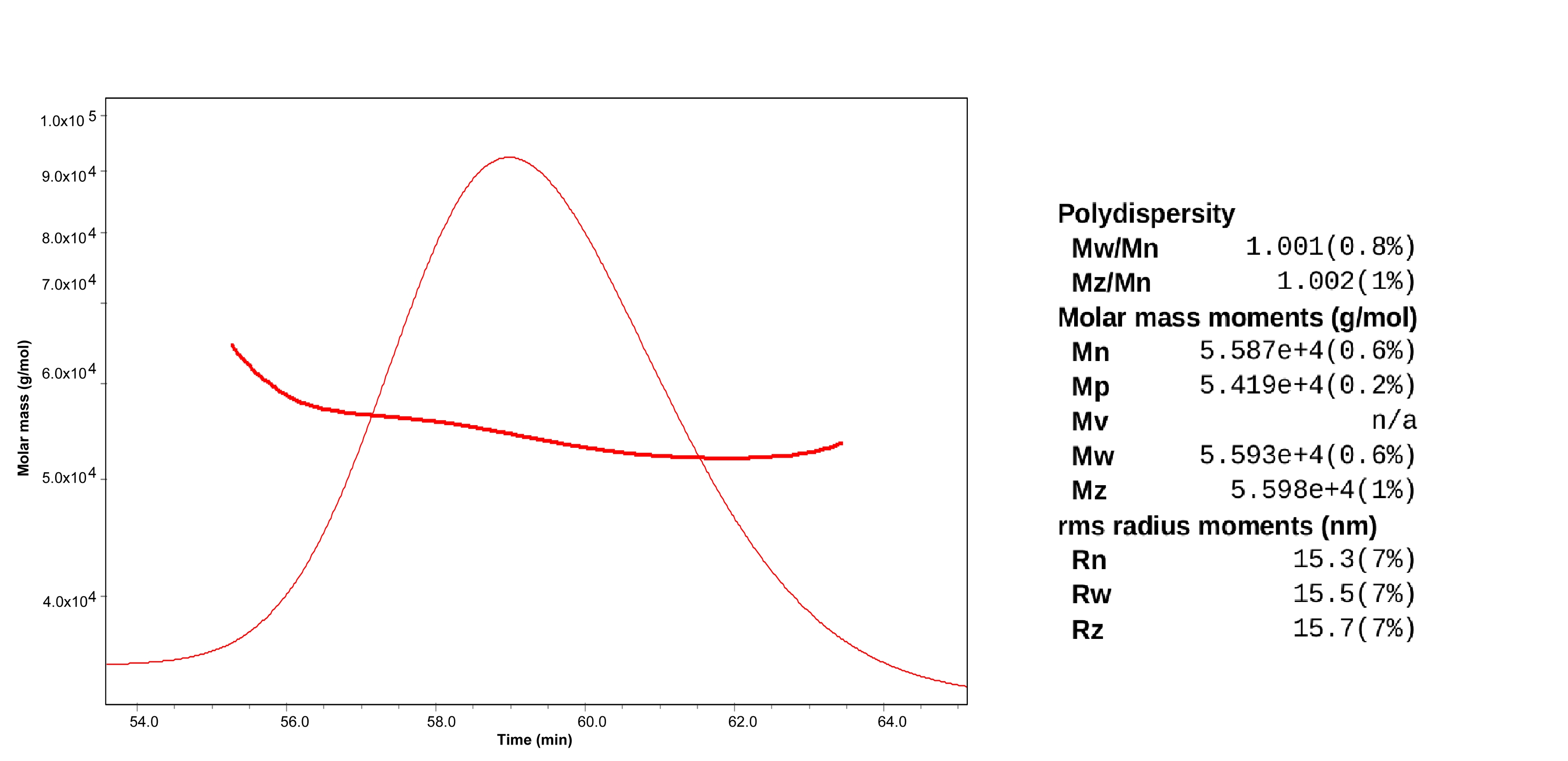

Supplement: Figure S1 — The oligomeric state analysis of the recombinantly expressed, purified Ms TLP1 has been determined by using static light scattering (SLS) in combination with size-exclusion chromatography. A Superdex 200 10/300 GL column (GE Healthcare) was used for the size-exclusion chromatography. The elution profile is provided by the UV signal (thin red line). The SLS signal is combined with the RI-signal for the calculation of the molar mass (thick red line) by the ASTRA program. (TIFF) [file pone.0041894.s001.tiff]

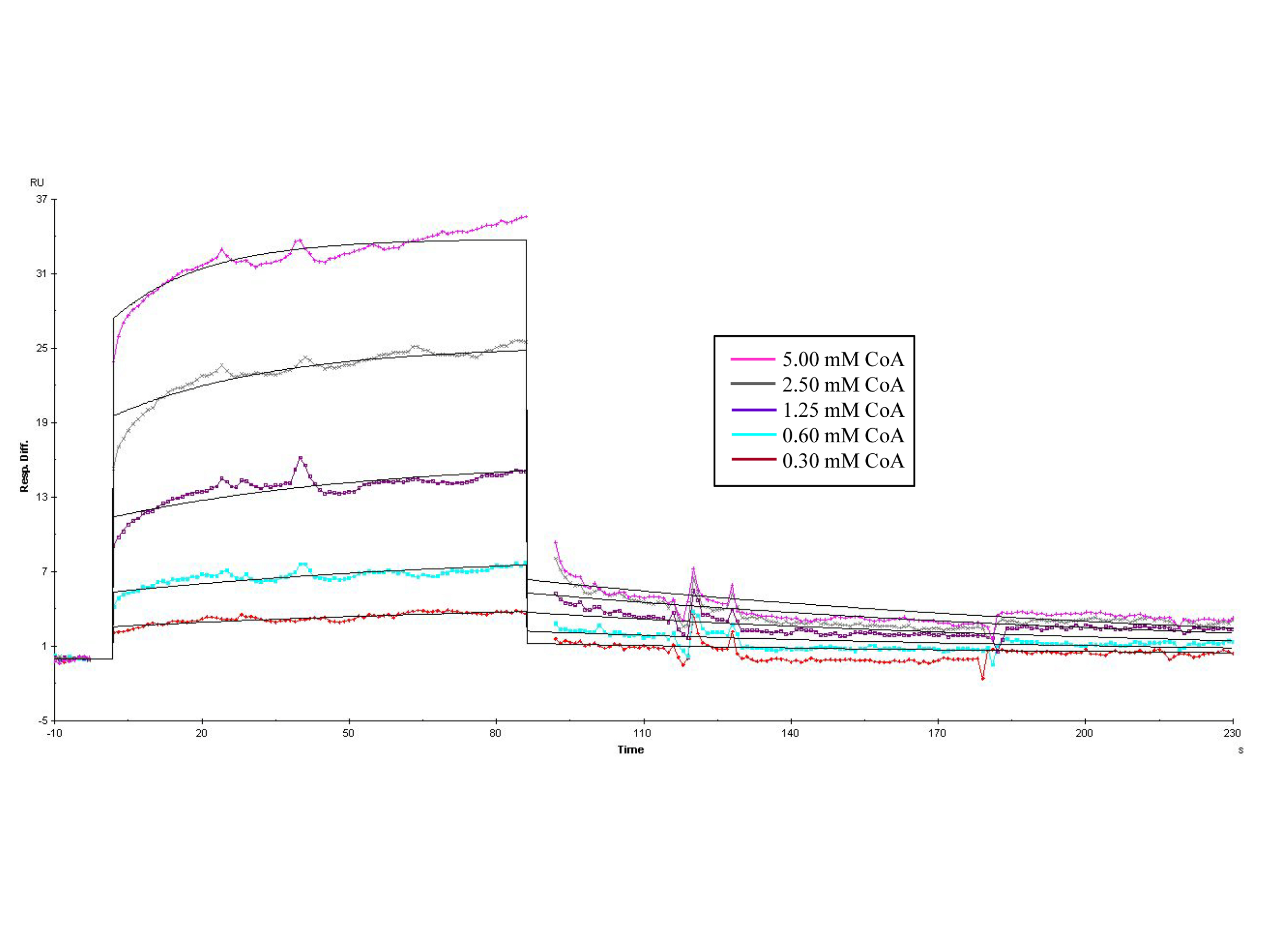

Supplement: Figure S2 — Kinetics of binding of CoA to Ms TLP1 determined using SPR. The SPR sensogram was obtained by flowing different CoA solutions (see inset) over the TLP1 immobilized sensor chip. The data show that CoA binds to TLP1 with a Kd in the millimolar range. (TIFF) [file pone.0041894.s002.tiff]
